# Multiplex indexing approach for the detection of DNase I hypersensitive sites in single cells

**DOI:** 10.1093/nar/gkab102

**Published:** 2021-03-08

**Authors:** Weiwu Gao, Wai Lim Ku, Lixia Pan, Jonathan Perrie, Tingting Zhao, Gangqing Hu, Yuzhang Wu, Jun Zhu, Bing Ni, Keji Zhao

**Affiliations:** Laboratory of Epigenome Biology, Systems Biology Center, National Heart, Lung and Blood Institute, NIH, Bethesda, MD, USA; Institute of Immunology of PLA, Third Military Medical University, Chongqing 400038, PR China; Department of Pathophysiology, College of High Altitude Military Medicine, Third Military Medical University, Chongqing 400038, PR China; Laboratory of Epigenome Biology, Systems Biology Center, National Heart, Lung and Blood Institute, NIH, Bethesda, MD, USA; Laboratory of Epigenome Biology, Systems Biology Center, National Heart, Lung and Blood Institute, NIH, Bethesda, MD, USA; Laboratory of Epigenome Biology, Systems Biology Center, National Heart, Lung and Blood Institute, NIH, Bethesda, MD, USA; Chongqing International Institute for Immunology, Chongqing 401338, PR China; Laboratory of Epigenome Biology, Systems Biology Center, National Heart, Lung and Blood Institute, NIH, Bethesda, MD, USA; Institute of Immunology of PLA, Third Military Medical University, Chongqing 400038, PR China; Laboratory of Epigenome Biology, Systems Biology Center, National Heart, Lung and Blood Institute, NIH, Bethesda, MD, USA; Institute of Immunology of PLA, Third Military Medical University, Chongqing 400038, PR China; Department of Pathophysiology, College of High Altitude Military Medicine, Third Military Medical University, Chongqing 400038, PR China; Laboratory of Epigenome Biology, Systems Biology Center, National Heart, Lung and Blood Institute, NIH, Bethesda, MD, USA

## Abstract

Single cell chromatin accessibility assays reveal epigenomic variability at *cis*-regulatory elements among individual cells. We previously developed a single-cell DNase-seq assay (scDNase-seq) to profile accessible chromatin in a limited number of single cells. Here, we report a novel indexing strategy to resolve single-cell DNase hypersensitivity profiles based on bulk cell analysis. This new technique, termed indexing single-cell DNase sequencing (iscDNase-seq), employs the activities of terminal DNA transferase (TdT) and T4 DNA ligase to add unique cell barcodes to DNase-digested chromatin ends. By a three-layer indexing strategy, it allows profiling genome-wide DHSs for >15 000 single-cells in a single experiment. Application of iscDNase-seq to human white blood cells accurately revealed specific cell types and inferred regulatory transcription factors (TF) specific to each cell type. We found that iscDNase-seq detected DHSs with specific properties related to gene expression and conservation missed by scATAC-seq for the same cell type. Also, we found that the cell-to-cell variation in accessibility computed using iscDNase-seq data is significantly correlated with the cell-to-cell variation in gene expression. Importantly, this correlation is significantly higher than that between scATAC-seq and scRNA-seq, suggesting that iscDNase-seq data can better predict the cellular heterogeneity in gene expression compared to scATAC-seq. Thus, iscDNase-seq is an attractive alternative method for single-cell epigenomics studies.

## INTRODUCTION

Cellular heterogeneity in gene expression, has been extensively studied through single-cell sequencing methods. For example, single-cell RNA sequencing (scRNA-seq) has revealed significant heterogeneity in primary glioblastomas (1). Also, increased levels of heterogeneity in these tumors are inversely correlated with survival, indicating that intratumor heterogeneity should be an essential clinical factor. Successful identification of regulators of this heterogeneity is critical to the development of new therapeutic drugs.

DNase I hypersensitivity of chromatin informs the chromatin states of *cis*-regulatory elements that govern the expression of target genes including master regulators (2–5). Cellular heterogeneity in gene expression has been linked to variation in chromatin accessibility (6), nucleosome organization (2) and long distance enhancer-promoter interactions (7); thus, measuring chromatin states at the single-cell level is of the utmost importance for understanding the molecular mechanisms of gene expression heterogeneity. Several single cell techniques were developed to measure chromatin accessibility, including scATAC-seq (3–5,8–10) by Tn5 chromatin tagmentation, scDNase-seq (6) by DNase I digestion for chromatin fragmentation, and scMNase-seq (2) by MNase detection of chromatin accessibility and nucleosome positions. The standard throughput of many of these methods is in the thousands of cells, and of these methods scATAC-seq has the highest cell throughputs; however, it is also known that DNA tagmentation bias exists in the use of Tn5 (11), which may affect the accuracy of the regulator prediction and cell-to-cell variation in accessibility, limiting its potential applications.

DNase I enzymes have different properties compared to Tn5 (12). However, due to a lack of development in combinational indexing strategies for scDNase-seq, its cell throughput is very low and thus its application in single-cell studies is limited. To address this limitation, we designed a novel indexing strategy, which avoids the use of expensive equipment for automation or microfluidics, to enable the analysis of >15 000 cells in a single experiment. This new strategy, termed indexing scDNase-seq (iscDNase-seq), involves barcoding the DNA ends with a combination of TdT terminal transferase and T4 DNA ligase. We applied it to assay single-cell DHSs from human white blood cells (WBC). Computational analysis of the assay results recovered expected cell types from the WBCs and inferred their underlying regulatory mechanisms in accessibility variation. By comparing our iscDNase-seq data with publicly available dscATAC-seq data (3–5,8–10) for B cells, T cells, NK cells and monocytes, we found that iscDNase-seq detects DHSs missed by scATAC-seq that have high sequence conservation and are associated with significant gene expression. Importantly, iscDNase-seq data can better predict the cellular heterogeneity in gene expression compared to scATAC-seq data. Thus, iscDNase-seq is an attractive alternative method for measuring single-cell chromatin accessibility.

## MATERIALS AND METHODS

### Experimental methods

#### iscDNase-seq method

Reagents and oligo sequences are listed in the Supplementary Materials S2 and S3. In the iscDNase-seq protocol (Supplementary Figure S1), cells were first crosslinked by two-step fixation and subjected to lysis and DNA digestion with DNase I on bulk cells. After removal of DNase I by several washes, bulk nuclei were aliquoted into 96 wells and barcode P7 adaptors were ligated to the chromatin DNA by the TdT&T4 ligation method. The samples were then pooled, diluted, and redistributed to 96 wells of a second plate with 30 nuclei to each well using a flow cytometry sorter. After reverse-crosslinking of DNA overnight at 65°C, a second barcode (well index) primer complementary to the P7 adapter, was introduced to the DNA template directly by one-cycle of polymerase chain reaction (PCR1). Then, all PCR1 products were pooled, ligated to P5 adaptor and re-amplified by PCR2 primers that introduced the third barcode (i5 index). Finally, all of PCR2 products were pooled and sequenced, with the expectation that most sequence reads bearing the same combination of barcodes will be derived from a single cell (estimated collision rate of ∼13% for experiments described here)

#### oligo sequence

Barcode P7 adaptor top (/5phos/ACACTGACGACATGGTTCTACANNNNNNNNAGATCGGAAGAGCACACGTCTGAACTCCAGTCAC/3SpC3/).

Barcode P7 adaptor bottom

(TGTAGAACCATGTCGTCAGTGTCCCCCCCC/3ddC/)

Well index primer (TACGGTAGCAGAGACTTGGTCTNNNNNNGTGACTGGAGTTCAGACGTGTGCTCTTCCG)

I5 index primer (AATGATACGGCGACCACCGAGATCTACACNNNNNNNNACACTCTTTCCCTACACGACGCT)

P7-cs2 primer (CAAGCAGAAGACGGCATACGAGATTACGGTAGCAGAGACTTGGTC*T)

P5 adaptor top (/5phos/GATCGGAAGAGCGTCGTGTAGGGAAAGAGTG)

P5 adaptor bottom (TCTTTCCCTACACGACGCTCTTCCGATCT)

#### Isolation of PBMC

Human healthy donor bloods were collected and defibrinated or heparinized in a EDTA sodium-treated tubes or bags for anticoagulant of blood by the NIH blood bank. The peripheral blood mononuclear cells (PBMC) were purified by the density centrifugation using Lymphocyte Separation Medium (Corning, catalog no. 45000-726).

#### Two-step crosslinking of cells

The isolated 50M of PBMC suspended in 50 ml PBS/MgCl_2_ were first fixed by adding 400 μl freshly prepared 0.25 M Disuccinimidyl glutarate (DSG, ThermoFisher Scientific, catalog no. 20593) and incubating at room temperature for 45 min with rotation (13). After three washes with PBS, the cells were suspended in culture medium DMEM supplemented with 10% FBS and further fixed by adding 1:15 volume of 16% (w/v) methanol-free formaldehyde solution (Thermo Fisher Scientific) and incubating at room temperature for 10 min (14). The reaction was terminated by adding a 1:10 volume of 1.25 M glycine and incubating at room temperature for 5 min. The fixed cells were collected by centrifugation at 1320 rpm for 7 min and washed with PBS. The fixed cells were stored in aliquots (1 × 10^6^ cells per tube) at −80°C until use.

#### DNase I digestion

The two-step fixed cells (1 × 10^6^) were suspended in 0.5 ml of RSB buffer (10 mM Tris–HCl pH 7.4, 10 mM NaCl, 3 mM MgCl_2_, 0.1% Triton X-100) and incubated for 10 min on ice. 50 units of DNase I were added to the cells, followed by incubation in 37°C water bath for 5 min to digest the chromatin (Pilot DNase I titration is needed (15)). The reaction was quenched by adding 10 μl 0.5 M EDTA to a final concentration of 10 mM. The cells were centrifuged at 1320 rpm for 5 min at 4°C. The supernatants were carefully removed by pipetting without disturbing the cell pellets. The pellets were washed three times using 1ml 1 × T4 ligase buffer (final 0.1% NP40) to remove the DNase I completely.

#### TdT&T4 ligation

The DNase I-digested cells were resuspended in nuclei resuspension buffer (328 μl H_2_O; 132 μl 10 mM dGTP; 66 μl 10 × T4 ligase buffer; 5.3 μl 10% NP40) and equally distributed to 96 wells of a 96-well plate. To add several Gs at the 3′ end of DNA and allow adaptor ligation, 2.5 μl of 10 μM barcode P7 adaptor were added into each well, followed by adding 5 μl of the enzyme dilution buffer (66 μl 10 × T4 ligase buffer; 330 μl H_2_O; 40 μl TdT enzyme; 13 μl T4 PNK; 78.75 μl T4 ligase) with gentle mixing (pipette up and down 5–7 times). TdT&T4 ligation is performed on the PCR machine for 30 min at 37°C with lid heating.

#### Pool and split

After TdT&T4 ligation, nuclei were pooled and re-suspended in 1ml PBS containing 0.1% NP40 and 3 μM DAPI (Invitrogen) for nuclei staining. After 5 min incubation at room temperature, the nuclei were counted under the DAPI fluorescent microscope and 30 nuclei were distributed, using a flow cytometry sorter, into each well of a 96-well plate containing 3 μl reverse-crosslink buffer (50 mM Tris–HCl, pH 8.0, 25 ng/ml Proteinase K, 0.1% NP40) mixed with 10 μl PBS containing 0.1% NP40. Up to six plates of cells were collected. The plates were sealed completely and incubated at 65°C overnight on PCR machine with lid heating. After reverse-crosslinking, add 2.5 μl of 2 μM well index primer and 15 μl of 2 × Phusion^®^ master mix (New England BioLabs, catalog no. M0531S) into each well for PCR1 amplification without DNA purification. The PCR1 was done under the following condition: 98°C, 3 min; followed by 12 cycles of 65°C, 30 s and 72°C, 30 s; one cycle of 72°C, 5 min. After PCR1, for each 96-well plate, all of the products were pooled and incubated with 96 μl of Exonuclease I (ThermoFisher Scientific, catalog no. EN0582) at 37°C for 30 min to degrade the excessive of well index primers. DNA was then purified by the MinElute^®^ Reaction Cleanup Kit (Qiagen, catalog no. 28206).

#### Library preparation and sequencing

A-tailing and P5 adaptor ligation were performed as described previously (16). After P5 adaptor ligation, library DNA is purified by the MinElute^®^ Reaction Cleanup Kit. PCR2 was performed by adding 15 μl DNA; 0.4 μl of 10 μM i5 primer; 0.4 μl of 10 μM p7-cs2 primer; 15.8 μl 2× Phusion Master Mix with the following condition: 98°C, 3 min; 57°C, 3 min; 72°C, 1 min; followed by 15 cycles of 98°C, 10 s; 65°C, 15 s and 72°C, 30 s; one cycle of 72°C, 5 min. The 220–600 bp fragments were isolated using the 2% E-Gel^®^ EX Agarose Gels (Invitrogen, cat #G401002) and purified using the QIAquick Gel Extraction kit (Qiagen). The concentration of the purified DNA was measured using Qubit dsDNA HS kit (Thermo Fisher Scientific). The paired-end 50–6–8–50 sequencing was performed using the Illumina MiSeq and HiSeq 3000.

### Data analysis

#### Demultiplexing and data analysis of iscDNase-seq libraries

The scripts for de-multiplexing and genome-wide mapping are available at https://github.com/wailimku/iscDNase-seq.git. Thirty single cells were sorted into each of the 480 wells by FACS and sent to sequencing after the library's preparation steps. All sequencing data was paired-end. The R2 reads contained the information of cell barcodes (Supplementary Material S2). For each well, R1 reads were mapped to the human reference genome (UCSC hg18) using Bowtie2 (17). Using the cell barcode information from R2 reads, we separated the mapped R1 reads into 96 sets corresponding to the 96 cell barcodes. Reads with mapping quality <10 were removed and duplicated reads were removed. For each well, in order to determine the sets of mapped reads among the 96 sets were from single cells, we ranked the 96 sets of mapped reads based on the total number of mapped reads in the sets. A set of reads were considered to be from single cells if they satisfied:

1. They were one of the top 25 ranked sets.
2. The total number of mapped reads in the set was >1,000.

The single cell statistics were in Supplementary Material S3. For further filtering the single cells, the merged peaks identified by bulk-cell DNase-seq data were downloaded from ENCODE. Totally, bulk cell DNase-seq libraries were downloaded from ENCODE (Supplementary Table S1). For each of the bulk-cell DNase-seq library, peaks were called using MACS2 (18), and peaks from all libraries were merged if they overlapped by at least 1 bp. Finally, 218 595 were identified for the bulk-cell DNase-seq data for human WBC. The width of peaks was fixed to be 1,000bp. A further filtering step was applied to the selected single cells by requiring that reads in single cell need to be >4000 and FRiP (fraction of reads in peaks defined by the bulk-cell DNase-seq data) of single cell need to be >0.15.

#### Examining the quality of iscDNase-seq data

All reads from single cells were pooled together and visualized via the WashU genome browser (19) together with the bulk-cell DNase-seq data. Peaks from the pooled single cells were identified using MACS (18) and their widths were fixed to be 500 bp. The overlap between peaks from the pooled single cells and the bulk-cell data were computed using the function ‘FindOverlap’ in the R package called GenomicRanges (20). The read density of pooled single cell and pooled bulk-cell data from the 18 bulk-cell libraries were calculated over the bulk-cell peaks. In particular, peaks with read density equal to 0 from either pooled single cell or bulk cells were removed in the calculation. The correlation between the read densities of pooled single cell and bulk cell was quantified by the Pearson Correlation.

#### Clustering analysis for the iscDNase-seq data

##### Expression matrix

First, we computed a read count matrix $R$, in which the columns correspond to cell and rows correspond to DHSs that were identified using pooled single cells. ${R_{ij}}$ indicates the number reads at the DHS site $i$ from the $j$th cell. For filtering the non-information DHSs, DHSs with total number of reads over all single cells <150 were filtered out.

##### A Latent Semantic Indexing (LSI) analysis

Similar to the previous studies (4,21), we applied latent semantic indexing (LSI) to the read count matrix to reduce the dimensions. To perform the LSI analysis, the read count matrix was normalized by term frequency inverse document frequency (TF-IDF) and then a singular-value decomposition (SVD) was performed on the normalized count matrix. By removing thefirst dimension component after SVD transformation, the inverse SVD transformation was applied, resulting in a normalized read count matrix $E^{\prime}$ in which rows correspond to DHSs and columns correspond to cells.

##### t-SNE visualization and clustering

We applied a t-SNE to the normalized read count matrix $E^{\prime}$. The position of single cells was visualized in the 2D t-SNE representative space. Single cells are labeled in two different ways. First, single cells were labeled according to the clusters they were from. Second, single cells were labeled according the annotation of cell types. DBSCAN was applied to the two-dimensional t-SNE representative space for clustering.

#### Generating heatmap for the cluster specific reads of iscDNase-seq data

##### Identifying cluster specific peaks

The normalized read count matrix $E^{\prime}$ was transformed to another normalized matrix $G$ in which rows correspond to DHSs and columns corresponds to clusters. In particular, ${G_{ij}}$ = mean (${E^{\prime}_{ik}})$ for all cell $k$ belonging to cluster $j$. Further, the fold-change of DHSs in each cluster was computed where fold change at peak *i* for cluster *k*$ = \ {\rm{min}}( {\frac{{{G_{ik}}}}{{{G_{ij}}}}} )$ for all $j\ = \ 1,..,4\ and\ \ne k$. For each cluster, we selected DHSs with fold-change >1.5. Finally, the heatmap of $E^{\prime}$ at the specific peaks were plotted.

##### TF motif analysis

For each cluster, AME (22) was applied to the specific peaks for identifying significant motifs, and the top 40 significant motifs were selected first by also requiring *P*-value <0.01. Then of that set, only motifs exclusive to one cluster were kept.

#### Comparing iscDNase-seq against dscATAC-seq

##### Peak calling

Peaks were identified using MACS calls (parameters: –format bed –nomodel –call-summits –nolambda –keep-dup) on each assay-cell type. Unique peak sets are equivalent to A ∩ B’ where A is the assay of interest and B is the other assay with both sets belonging to the same cell type of either single cell or bulk assays. Unique intersecting peak sets are equivalent to taking the intersection between two unique peak sets where one belongs to single cells and the other belongs to bulk cells. These set operations are used to yield a refined set of peaks specific to a single cell assay that are also found in the bulk assay with the same digestion enzyme but not in other assays that use different enzymes.

##### Conservation scores

We compared unique intersecting peak sets by constructing average conservation score profiles for them. For each peak in a peak set, the average phastCons score was plotted at single bp resolution.

##### Enrichment analysis

We compared unique intersecting peak sets by finding the expression of their peaks’ nearest genes within 2.5 kb. Expression data was gathered from GEO (Supplementary Table S1) and the reads per kilobase per million mapped reads was calculated using rpkmforgenes.py^24^. Peaks were then annotated using ChIPseeker (23)with the gene expression data from rpkmforgenes.py.

#### Correlation between cell-to-cell variation in gene expression and accessibility

Coefficient of variation scores were calculated for peak accessibility and gene expression, where the gene expression data came from 10X Genomics. For annotating peaks with TSS, ChIPseeker (23) was used with a 20 kb range, and genes and peaks with no mapped reads were filtered out.

## RESULTS

### TdT terminal transferase and T4 DNA ligase-mediated barcoding strategy

The iscDNase-seq procedure is illustrated in Supplementary Figures S1 and S2. Following DNase I digestion of cells crosslinked with formaldehyde and disuccinimidyl glutarate (DSG), several dGs are added to the DNA ends by the activity of TdT in the presence of T4 DNA ligase and oligo-dC barcode adaptors in a 96-well plate (Supplementary Figure S1). Following base-paring with the oligo-dGs at the DNA ends, the oligo-dC barcode adaptors are ligated to the DNA ends by T4 DNA ligase. The cells are then pooled from 96 wells and aliquoted into new 96-well plates with 30 cells per well by flow cytometry sorting followed by two consecutive rounds of PCR amplification and indexing of DHS DNA (Supplementary Figure S1). The combination of three rounds of barcoding and indexing enables detection of over 15 000 cells in a single experiment.

**Figure 1.**
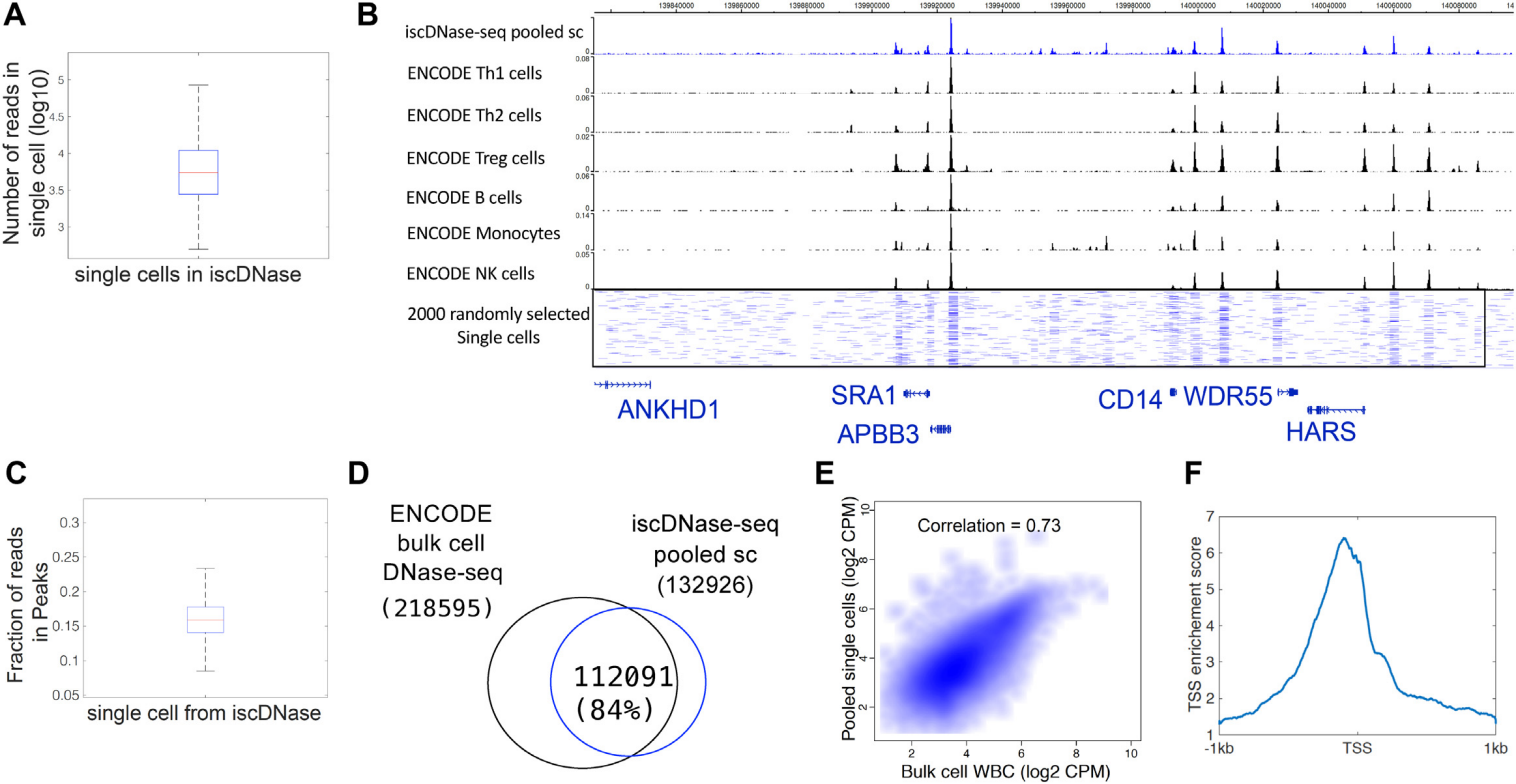
iscDNase-seq detects open chromatin regions in single cells. (**A**) A box plot to show the number of unique reads in single cells. (**B**) A genome browser snapshot showing chromatin accessibility detected by the pooled iscDNase-seq data and ENCODE bulk cell DNase-seq data for different immune cell types. The top track referred to the pooled iscDNase-seq data for human white blood cells. The other tracks, from the top to the bottom, referred to the ENCODE bulk cell DNase-seq data for Th1, Th2, Treg, B cells, monocytes, and NK cells, respectively. (**C**) A box plot to show the Fraction of reads in Peaks (FRiP) in single cells. (**D**) A Venn diagram showing the overlap between the DHSs obtained from the ENCODE DNase-seq data and the pooled single cell DNase-seq data. (**E**) A scatter plot showing the correlation between the read density of the bulk cell DNase-seq and pooled single cell DNase-seq at the DHSs. The correlation was computed using Pearson Correlation. (**F**) A TSS plot showing the TSS enrichment score of the pooled iscDNase-seq data.

We first applied iscDNase-seq to WBCs purified from human blood to detect open chromatin regions at single cell resolution. Using a cutoff to filter cells with less than 1000 reads and a fraction of reads in peaks (FRiP) smaller than 15%, we detected ∼15 000 single cells and 10 000 reads per cell on average in a single experiment (Figure 1A). Using a more stringent filtering criterion where a cell must have at least 4000 reads resulted in ∼10 000 single cells and 12 000 reads on average (Supplementary Figure S3A and S3B). The read statistics of single cells was shown in Supplementary Material S1. To test potential doublet formation by random collision between any two cells, human WBCs and mouse splenocytes mixed, cross-linked, subjected to DNase I digestion and processed for library construction. From the sequencing data, we observed a collision rate of approximately 13% (Supplementary Figure S3C), which was similar to a previous barcoding strategy for single-cell ATAC-seq (21). The genome browser snapshots (Figure 1B) show highly consistent profiles between the pooled single-cell and bulk cell ENCODE DNase-seq data. The mean of Fraction of reads in peaks is ∼0.16 (Figure 1C). We detected 218 595 and 132 926 DHSs from the bulk cell ENCODE data and the pooled single cell data, respectively, in which 112 091 (84%) overlapped (Figure 1D). The read densities of the pooled cells and the ENCODE data were highly correlated (Figure 1E). Also, the pooled single cell data showed enrichment around the transcription start site (TSS) with the enrichment score of 6 (Figure 1F).

All of these results together suggest that the iscDNase-seq method can effectively detect open chromatin regions in WBC.

### iscDNase-seq data accurately cluster sub-types of cells in WBC

**Figure 2.**
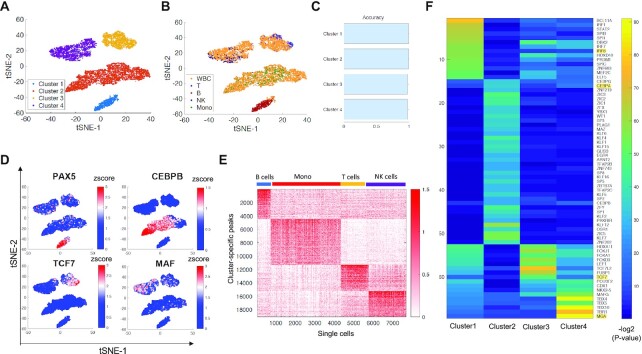
iscDNase-seq detects different sub cell types in human white blood cells and their specific regulatory regions. (**A**) A t-SNE visualization of cells with annotation of cells using the cluster information. (**B**) A t-SNE visualization of cells using the cell type information including the human WBCs, sorted B cells, sorted T cells, sorted NK cells, and sorted monocytes. (**C**) A bar plot showing the accuracy of cell clusters. (**D**) A t-SNE visualization of cells with the accessibility of selected TF genes. The color level indicates the zscore of accessibility across all the cells. Four TF genes were selected including (top left) PAX5, (top right) CEBPB, (bottom left) TCF7 and (bottom right) MAF. (**E**) The cluster-specific peaks show distinct enrichment in different cell types. A heatmap showing the z-score of the normalized read count at the specific peaks for each cluster. (**F**) Key transcription factor motifs enriched in the cluster-specific DHS peaks. Motif enrichment analysis was performed for each group of top specific peaks. The 80 most significant motifs were selected for each cluster. We eliminated those motifs that existed in more the one cluster. A heatmap was shown for the –log (*P*-value) for these TF motifs in each cluster.

Human WBCs contain T cells, NK cells, monocytes, and B cells. To benchmark cell cluster annotations, we applied iscDNase-seq to human CD4 T cells, B cells, NK cells, and monocytes that were purified by flow cytometry sorting. Using the same filtering strategy as the human WBCs, we obtained 699 B cells, 3590 monocytes, 1421 T cells, and 1923 NK cells. To cluster the single cells from each specific cell type, we first calculated read counts in the DHSs identified from the pooled single cell data for each of the sorted cell types and whole WBCs. Next, we applied the Latent Semantic Indexing method to normalize the data. Finally, the dimensionality reduction t-SNE was directly applied to the normalized read count matrix (Materials and Methods). Finally, the cluster results were visualized along with annotations of the known cell types and clusters (Figure 2A and B). The clustering analysis of WBCs revealed four clusters of cells (Figure 2A). The sorted B cells, T cells, NK cells and Monocytes were clearly clustered separately (Figure 2B). Comparison between the unsupervised and annotated clusters in Figure 2B suggests that clusters 1, 2, 3 and 4 belonged to B cells, Monocytes, T cells and NK cells, respectively. In order to evaluate the cluster annotations, we defined accuracy as the purity of a cluster or the largest fraction of one of the sorted cell types in a cluster. For example, the fraction of sorted B cells in cluster 1 is close to 100%, while the fractions of other sorted cell types are near zero; thus, cluster 1 cells are more likely to be annotated as B cells, and its cluster accuracy is close to 100%. We found that the cluster accuracies for clusters 1, 2, 3 and 4, which corresponded to B cells, Monocytes, T cells, and NK cells, were all >97% (Figure 2C). Within the human WBCs, there were about 47% monocytes, 19% T cells, 25% NK cells and 9% B cells. Overall, the iscDNase-seq data successfully clustered the four types of immune cells in human WBCs, which indicates that iscDNase-seq is able to identify cell type specific DHSs that can be used in downstream clustering.

Next, we examined whether any clusters were results of cell doublet formation. The reads per cell were visualized in the tSNE plots (Supplementary Figure S4A), and the results showed that the cells with extremely high read numbers did not aggregate in any one particular cluster, suggesting that the formation of potential doublets did not affect the clustering results. Furthermore, by examining the accessibility of several genes encoding cell-type specific TFs in the cells of the different clusters, we observed that cell-type specific TF genes (PAX5 for B cells, CEBPB for monocytes, TCF7 for T cells, and MAF for NK cells) exhibited the highest accessibility in the clusters annotated to be the same cell types that express the gene (Figure 2D).

Next, we examined whether we could identify cell type specific regulatory regions using our iscDNase-seq data. To do this, we detected the marker peaks that can distinguish each cluster from the other clusters (Methods). As shown in Figure 2E, the cluster-specific peaks have the highest normalized read counts in the specifically annotated cell types. To identify potential transcription factors that are associated with the cluster-specific peaks, we detected enriched motifs using AME (24). For each cluster, the top 40 significant motifs were selected first, and then of that set, only motifs exclusive to one cluster were kept (Figure 2F). We found that the set of enriched motifs in each cluster included target motifs for specific transcription factors known to be critical to the cell types that the clusters belonged to. For example, the IRF8 motif, which is specific to B cells (25), was enriched in cluster 1, which corresponds to B cells; the CEBPA motif, which is specific to Monocytes (26), was enriched in cluster 2, which corresponds to Monocytes; the TCF7 motif, which is critical to T cells (27), was enriched in cluster 3, which corresponds to T cells; and the MGA motif, which is specific to NK cells (28,29), was enriched in cluster 4, which corresponds to NK cells. To further confirm whether these TFs were specifically expressed in the corresponding cell types, their gene expression levels in the bulk cell data were examined and the four TFs were found to be specifically expressed in the corresponding cell types (Supplementary Figure 4B). These results suggest that iscDNase-seq is an efficient method to detect regulatory regions that are associated with cell-type specific TFs.

### iscDNase-seq and scATAC-seq reveal both common and distinct information in WBCs

**Figure 3.**
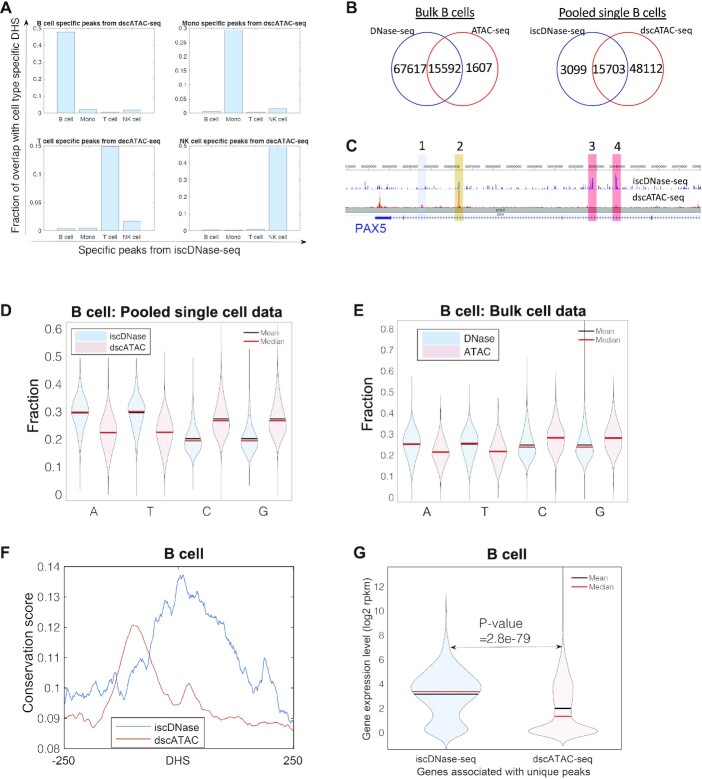
iscDNase-seq predicts functional open chromatin regions. (**A**) A bar plot showing the overlap between the cell type specific peaks from dscATAC-seq and the cell type specific peaks from the iscDNase-seq. Each subplot refers to the comparison between the cell type specific peaks from dscATAC-seq in one cell type with the cell type specific peaks from iscDNase-seq in four cell types. (**B**) Venn diagrams showing the overlap between peak sets from bulk DNase-seq and bulk ATAC-seq in B cells (left) and the overlap between the peak sets from iscDNase-seq and dscATAC-seq in B cells (right). (**C**) A Genome Browser track showing similarities and differences between the iscDNase-seq and dscATAC-seq datasets at the PAX5 gene locus in B cells. (**D**) A violin plot showing the fraction of nucleotides (A, T, C and G) at the unique peaks from iscDNase-seq and dscATAC-seq for B cells. (**E**) A violin plot showing the fraction of nucleotides (A, T, C and G) at the unique peaks from bulk cell DNase-seq and bulk cell ATAC-seq for B cells. (**F**) Sequence conservation scores from B cells for the unique iscDNaseq peaks and unique dscATAC-seq peaks. The unique peaks detected by iscDNase-seq are more likely conserved peaks than those uniquely detected by dscATAC-seq. (**G**) A violin plot showing the gene expression levels in B cells of genes associated with unique iscDNase-seq, unique dscATAC-seq peaks.

scATAC-seq and iscDNase-seq use different enzymes (Tn5 or DNase I) to probe chromatin accessibility, and thus iscDNase-seq may reveal information that is not recognized by scATAC-seq. To test this idea, we downloaded the recent single cell ATAC-seq data (dscATAC-seq) (10) for B cells, monocytes, T cells, and NK cells (10). For both dscATAC-seq and iscDNase data, the cell-type specific peaks were identified using MACS with a peak width setting of 500 bp. By comparing the cell-type specific peaks from iscDNase-seq with those from dscATAC-seq, we found that peaks from iscDNase-seq were highly overlapped with the peaks from dscATAC-seq only when they were from the same cell type (Figure 3A). This indicates that both assays are able to identify cell-specific open chromatin regions. Global analysis of the accessible sites in single cell and bulk cell assays revealed that a non-trivial fraction of the open regions was detected only by the DNase- or Tn5-related assays (Figure 3B, Supplementary Figure S5). For example, iscDNase-seq and dscATAC-seq found 3,099 and 48,112 peaks distinct from the other assay in B cells, respectively (Figure 3B, right panel). Visual inspection of the accessible sites on Genome Browser snapshots revealed distinct sites detected by iscDNase-seq and dscATAC-seq across gene loci. For example, iscDNase-seq and scATAC-seq detected same as well as distinct sites across the PAX5 gene locus in B cells (Figure 3C). While Site 2 was highly accessible in both assays (brown), Sites 3 and 4 were preferentially detected by iscDNase-seq (red) and Site 1 was preferentially detected by dscATAC-seq (blue).

To examine the functional significance of unique sites detected by iscDNase-seq versus dscATAC-seq, we first analyzed the gene ontology terms associated with the unique sites. We found that the enriched GO terms for the unique sites detected by iscDNase-seq and dscATAC-seq were very different (Supplementary Figure S6). The GO terms associated with unique iscDNase-seq peaks include histone modifications (B cells), myeloid cell differentiation (Monocytes), chromatin organization and NF-kappaB signaling (T cells), NF-kappaB signaling (NK cells). Many of these GO terms are related to immune functions. However, the GO terms associated with unique dscATAC-seq peaks include canonical WTN signaling pathway and kidney epithelium development (B cells), embryonic organ morphogenesis and skeletal system morphogenesis (Monocytes), axon guidance and neuron projection guidance (T cells and NK cells). These terms are not associated with immune functions. From these results, it appears that the unique peaks from the iscDNase-seq datasets are more likely to be associated with cell-specific functions of the underlying cells. Thus, the unique peaks from the iscDNase-seq date sets may be a better predictor of cell-specific enhancers than the unique dscATAC-seq peaks.

Next, we compared the nucleotide compositions of unique sites detected by iscDNase-seq and dscATAC-seq. We observed that the unique iscDNase-seq sites were more likely to be AT-rich while the unique dscATAC-seq peaks were more likely to be CG-rich (Figure 3D and Supplementary Figure S7). These trends were also observed in the unique peaks from the bulk cell DNase-seq and ATAC-seq data (Figure 3E and Supplementary Figure S7). It has been suggested that AT-rich regions were more related to the cell type (30). These results motivated the hypothesis that the unique iscDNase-seq peaks are more likely to contribute to transcriptional regulation than the unique dscATAC-seq peaks do.

To test this hypothesis, we compared their level of sequence conservation as sequence conservation is often an indicator of functional element. By retrieving the average phastCons conservation scores (31) of the unique iscDNase-seq and dscATAC-seq sites, we observed that the unique DNase-seq sites were more likely to have a conserved region around the center of the sites, while the unique dscATAC-seq peaks have a lower conserved region away from the center of the sites (Figure 3F and Supplementary Figure S8). Next, we identified the genes that are located near either a unique iscDNase-seq peak or a unique dscATAC-seq peak (Materials and Methods) and compared the expression levels of the two gene groups. The analysis revealed that the genes located near unique iscDNase-seq sites showed significantly higher expression levels than those located near unique dscATAC-seq sites (Figure 3G and Supplementary Figure S9). These results suggest that the unique iscDNase-seq peaks may be more likely to contribute to transcriptional regulation than the unique dscATAC-seq peaks do.

### iscDNase-seq provide better prediction of cellular heterogeneity in gene expression compared to scATAC-seq

**Figure 4.**
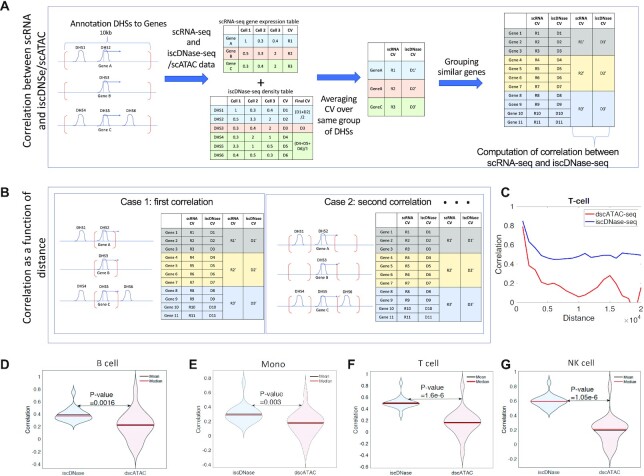
The cell-to-cell variation in DHS detected by iscDNase-seq is highly correlated with variation in gene expression. (**A**) A schematic diagram showing the calculation for the correlation between cell-to-cell variation in gene expression and accessibility. First, Genes are annotated to the nearest DHSs located within the selected genomic regions enclosed by the red brackets. Second, we computed the density table and gene expression table for dscATAC-seq/iscDNase-seq and scRNA-seq, respectively. Also, for each gene and DHSs, we computed the coefficient of variation. Third, more than one DHS may be annotated to a gene. If it was the case, an average coefficient of variation (CV) was taken over DHSs which were annotated to the same gene. Forth, 20 genes were grouped in a group based on their CV in accessibility. Fifth, we computed the averaged CV for each group of genes and each assay. Spearman correlation was computed between CV obtained from scRNA-seq and iscDNase-seq/dscATAC-seq over the groups of genes. (**B**) By varying the selection of the genomic regions enclosed by the red brackets, multiple correlation coefficients are obtained. In particular, the DHS regions closest to the TSSs were first selected. Then the DHS regions with increasing distance from the TSSs were selected. (**C**) The correlation between cell-to-cell variation in gene expression and accessibility for T cells were plotted as a function of distance, in which distance refers to the distance between the selected genomics regions and the closest TSSs. Correlation for both dscATAC-seq (red) and iscDNase-seq (blue) were computed. (**D**) A violin plot for correlation between cell-to-cell variation in gene expression and accessibility for B cells for both dscATAC-seq and iscDNase-seq were plotted. (**E**) A violin plot for correlation between cell-to-cell variation in gene expression and accessibility for monocytes for both dscATAC-seq and iscDNase-seq were plotted. (**F**) A violin plot for correlation between cell-to-cell variation in gene expression and accessibility for T cells for both dscATAC-seq and iscDNase-seq were plotted. (**G**) A violin plot for correlation between cell-to-cell variation in gene expression and accessibility for NK cells for both dscATAC-seq and iscDNase-seq were plotted.

One major goal of performing single-cell experiments is to examine the cellular heterogeneity. Elucidating the relationship between cell-to-cell variation in different omics layers is critical for identifying the origins of cellular heterogeneity and understanding how different omics layers interact. Previous studies reported that cell-to-cell variation in accessibility is positively correlated with that in gene expression. However, it is not clear whether the degree of difference in detecting accessibility could affect this correlation. To address this question, we computed the correlation between iscDNase-seq or dscATAC-seq with scRNA-seq as described in Figure 4A and B.

The strategy of calculating the correlation between iscDNase-seq or dscATAC-seq with scRNA-seq is described below (Figure 4A and B). DHSs were annotated to a gene if the distance between them is shorter than a threshold (e.g. 10 kb). Therefore, while computing the cell-to-cell variation in gene expression, the corresponding cell-to-cell variation in accessibility can also be computed. Note that the cell-to-cell variation is characterized by the coefficient of variation. Also, genes are aggregated into different groups based on the ranked CV in accessibility. Each group of genes are assigned with the average cell-to-cell variation in both gene expression and accessibility. Finally, the correlation between cell-to-cell variation in gene expression and accessibility over the groups of genes (Figure 4A) is computed.

It is possible that either of the assays detects the more precise accessibility of the open chromatin regions at different distances away from TSSs. Therefore, genome regions that are 20 kb downstream and upstream of TSSs are divided into bins with equal bin size of 1,000 bp. For each assay, we computed multiple correlation coefficients between the variation in accessibility and gene expression, using different annotations of DHSs to TSS based on the consideration of different bins. In each calculation, only bins that have the same distance away from TSSs were considered. Finally, we obtained a set of correlation coefficients which refer to bins that are located away from TSSs with different distances (Figure 4B). DHSs that are further away from TSSs is expected to have lower impact to the gene expression of the TSSs. Indeed, we observed that the correlation between cell-to-cell variation in accessibility and gene expression decrease, for both iscDNase-seq and dscATAC-seq, when the distance between the considered DHSs and TSSs increases (Figure 4C). However, the correlation between iscDNase-seq and scRNA-seq is significantly higher than that between dscATAC-seq and scRNA-seq through all distances (Figure 4C). Furthermore, the variation in accessibility of iscDNase-seq peaks annotated to TSS is significantly better correlated with variation in gene expression than the variation measured by dscATAC-seq peaks (Figure 4D–G).

## DISCUSSION

We previously demonstrated scDNase-seq is a sensitive method for detecting genome-wide DHSs in very small number of cells or single-cells (6). Furthermore, cell-to-cell variation in chromatin accessibility calculated using single-cell DHS data generated by scDNase-seq was highly correlated with that of gene expression based on scRNA-seq data (6). In this study, we designed a new strategy, iscDNase-seq, to dramatically improve the throughput of single-cells that can be analyzed in one experiment. iscDNase-seq is capable of analyzing tens of thousands of single-cells in one experiment, 100-fold improvement compared with the current scDNase-seq method, without the need of expensive and sophisticated equipment and accessible to most molecular biology laboratories.

Although both ATAC-seq and DNase-seq provide information on chromatin accessibility, recent studies found that DNase-seq and ATAC-seq can detect different chromatin open regions and DNase-seq is more likely to detect enhancer regions compared to ATAC-seq (3,11,12), suggesting that iscDNase-seq and single cell ATAC-seq assays may detect different properties of chromatin. Although our results from comparing the iscDNase-seq data and single cell ATAC-seq data indicated that the data generated using iscDNase-seq protocol has higher background and lower enrichment compared to current dscATAC-seq protocol, the DHS regions uniquely detected by iscDNase-seq showed higher sequence conservation scores than those uniquely detected by scATAC-seq. Furthermore, we demonstrate that the genes located near DHSs uniquely detected by iscDNase-seq exhibited higher expression levels than the genes located near DHSs uniquely detected by single cell ATAC-seq assays. These results indicated that iscDNase-seq is more likely to detect functional elements required for cell-specific gene expression than the single cell ATAC-seq assays do. Consistent with this, we found that the correlation between the cell-to-cell variations in gene expression and DHSs detected by iscDNase-seq is also significantly higher than that between the cell-to-cell variations in gene expression and DHSs detected by single cell ATAC-seq assays. All these results together suggest that iscDNase-seq is an attractive alternative single cell method for single-cell epigenomics studies.

## DATA AVAILABILITY

The iscDNase-seq data are available from GSE156017. The code can be downloaded from https://github.com/wailimku/iscDNase-seq.git.

## Supplementary Material

gkab102_Supplemental_Files

## References

[1] Patel A.P. , Tirosh I., Trombetta J.J., Shalek A.K., Gillespie S.M., Wakimoto H., Cahill D.P., Nahed B.V., Curry W.T., Martuza R.L. et al. Single-cell RNA-seq highlights intratumoral heterogeneity in primary glioblastoma. Science. 2014; 344:1396–1401. 24925914 10.1126/science.1254257 PMC4123637

[2] Lai B. , Gao W., Cui K., Xie W., Tang Q., Jin W., Hu G., Ni B., Zhao K. Principles of nucleosome organization revealed by single-cell micrococcal nuclease sequencing. Nature. 2018; 562:281–285. 30258225 10.1038/s41586-018-0567-3 PMC8353605

[3] Mezger A. , Klemm S., Mann I., Brower K., Mir A., Bostick M., Farmer A., Fordyce P., Linnarsson S., Greenleaf W. High-throughput chromatin accessibility profiling at single-cell resolution. Nat. Commun. 2018; 9:3647. 30194434 10.1038/s41467-018-05887-x PMC6128862

[4] Chen X. , Miragaia R.J., Natarajan K.N., Teichmann S.A. A rapid and robust method for single cell chromatin accessibility profiling. Nat. Commun. 2018; 9:5345. 30559361 10.1038/s41467-018-07771-0 PMC6297232

[5] Cusanovich D.A. , Hill A.J., Aghamirzaie D., Daza R.M., Pliner H.A., Berletch J.B., Filippova G.N., Huang X., Christiansen L., DeWitt W.S. et al. A single-cell atlas of in vivo mammalian chromatin accessibility. Cell. 2018; 174:1309–1324. 30078704 10.1016/j.cell.2018.06.052 PMC6158300

[6] Jin W. , Tang Q., Wan M., Cui K., Zhang Y., Ren G., Ni B., Sklar J., Przytycka T.M., Childs R. et al. Genome-wide detection of DNase I hypersensitive sites in single cells and FFPE tissue samples. Nature. 2015; 528:142–146. 26605532 10.1038/nature15740 PMC4697938

[7] Ren G. , Jin W., Cui K., Rodrigez J., Hu G., Zhang Z., Larson D.R., Zhao K. CTCF-Mediated enhancer-promoter interaction is a critical regulator of cell-to-cell variation of gene Expression. Mol. Cell. 2017; 67:1049–1058. 28938092 10.1016/j.molcel.2017.08.026 PMC5828172

[8] Buenrostro J.D. , Wu B., Litzenburger U.M., Ruff D., Gonzales M.L., Snyder M.P., Chang H.Y., Greenleaf W.J. Single-cell chromatin accessibility reveals principles of regulatory variation. Nature. 2015; 523:486–490. 26083756 10.1038/nature14590 PMC4685948

[9] Satpathy A.T. , Saligrama N., Buenrostro J.D., Wei Y., Wu B., Rubin A.J., Granja J.M., Lareau C.A., Li R., Qi Y. et al. Transcript-indexed ATAC-seq for precision immune profiling. Nat. Med. 2018; 24:580–590. 29686426 10.1038/s41591-018-0008-8 PMC5948148

[10] Lareau C.A. , Duarte F.M., Chew J.G., Kartha V.K., Burkett Z.D., Kohlway A.S., Pokholok D., Aryee M.J., Steemers F.J., Lebofsky R. et al. Droplet-based combinatorial indexing for massive-scale single-cell chromatin accessibility. Nat. Biotechnol. 2019; 37:916–924. 31235917 10.1038/s41587-019-0147-6 PMC10299900

[11] Li Z. , Schulz M.H., Look T., Begemann M., Zenke M., Costa I.G. Identification of transcription factor binding sites using ATAC-seq. Genome Biol. 2019; 20:45. 30808370 10.1186/s13059-019-1642-2 PMC6391789

[12] Karabacak Calviello A. , Hirsekorn A., Wurmus R., Yusuf D., Ohler U. Reproducible inference of transcription factor footprints in ATAC-seq and DNase-seq datasets using protocol-specific bias modeling. Genome Biol. 2019; 20:42. 30791920 10.1186/s13059-019-1654-y PMC6385462

[13] Tian B. , Yang J., Brasier A.R. Two-step cross-linking for analysis of protein–chromatin interactions. Methods Mol. Biol. 2012; 809:105–120. 22113271 10.1007/978-1-61779-376-9_7 PMC4148016

[14] Kidder B.L. , Hu G., Zhao K. ChIP-Seq: technical considerations for obtaining high-quality data. Nat. Immunol. 2011; 12:918–922. 21934668 10.1038/ni.2117 PMC3541830

[15] Cooper J. , Ding Y., Song J., Zhao K. Genome-wide mapping of DNase I hypersensitive sites in rare cell populations using single-cell DNase sequencing. Nat. Protoc. 2017; 12:2342–2354. 10.1038/nprot.2017.099 PMC11005227 29022941

[16] Ku W.L. , Nakamura K., Gao W., Cui K., Hu G., Tang Q., Ni B., Zhao K. Single-cell chromatin immunocleavage sequencing (scChIC-seq) to profile histone modification. Nat. Methods. 2019; 16:323. 30923384 10.1038/s41592-019-0361-7 PMC7187538

[17] Langmead B. , Salzberg S.L. Fast gapped-read alignment with Bowtie 2. Nat. Methods. 2012; 9:357–359. 22388286 10.1038/nmeth.1923 PMC3322381

[18] Zhang Y. , Liu T., Meyer C.A., Eeckhoute J., Johnson D.S., Bernstein B.E., Nusbaum C., Myers R.M., Brown M., Li W. et al. Model-based analysis of ChIP-Seq (MACS). Genome Biol. 2008; 9:R137. 18798982 10.1186/gb-2008-9-9-r137 PMC2592715

[19] Zhou X. , Maricque B., Xie M., Li D., Sundaram V., Martin E.A., Koebbe B.C., Nielsen C., Hirst M., Farnham P. et al. The human epigenome browser at washington university. Nat. Methods. 2011; 8:989–990. 22127213 10.1038/nmeth.1772 PMC3552640

[20] Lawrence M. , Huber W., Pages H., Aboyoun P., Carlson M., Gentleman R., Morgan M.T., Carey V.J. Software for computing and annotating genomic ranges. PLoS Comput. Biol. 2013; 9:e1003118. 23950696 10.1371/journal.pcbi.1003118 PMC3738458

[21] Cusanovich D.A. , Daza R., Adey A., Pliner H.A., Christiansen L., Gunderson K.L., Steemers F.J., Trapnell C., Shendure J. Multiplex single cell profiling of chromatin accessibility by combinatorial cellular indexing. Science. 2015; 348:910–914. 25953818 10.1126/science.aab1601 PMC4836442

[22] McLeay R.C. , Bailey T.L. Motif enrichment analysis: a unified framework and an evaluation on ChIP data. BMC Bioinformatics. 2010; 11:165. 20356413 10.1186/1471-2105-11-165 PMC2868005

[23] Yu G. , Wang L.G., He Q.Y. ChIPseeker: an R/Bioconductor package for ChIP peak annotation, comparison and visualization. Bioinformatics. 2015; 31:2382–2383. 25765347 10.1093/bioinformatics/btv145

[24] Heinz S. , Benner C., Spann N., Bertolino E., Lin Y.C., Laslo P., Cheng J.X., Murre C., Singh H., Glass C.K. Simple combinations of lineage-determining transcription factors prime cis-regulatory elements required for macrophage and B cell identities. Mol. Cell. 2010; 38:576–589. 20513432 10.1016/j.molcel.2010.05.004 PMC2898526

[25] Mookerjee-Basu J. , Kappes D.J. New ingredients for brewing CD4(+)T (cells): TCF-1 and LEF-1. Nat. Immunol. 2014; 15:593–594. 24940944 10.1038/ni.2927

[26] Feinberg M.W. , Wara A.K., Cao Z., Lebedeva M.A., Rosenbauer F., Iwasaki H., Hirai H., Katz J.P., Haspel R.L., Gray S. et al. The Kruppel-like factor KLF4 is a critical regulator of monocyte differentiation. EMBO J. 2007; 26:4138–4148. 17762869 10.1038/sj.emboj.7601824 PMC2230668

[27] Simonetta F. , Pradier A., Roosnek E. T-bet and eomesodermin in NK cell development, maturation, and function. Front. Immunol. 2016; 7:241. 27379101 10.3389/fimmu.2016.00241 PMC4913100

[28] Cobaleda C. , Schebesta A., Delogu A., Busslinger M. Pax5: the guardian of B cell identity and function. Nat. Immunol. 2007; 8:463–470. 17440452 10.1038/ni1454

[29] Wang H. , Lee C.H., Qi C., Tailor P., Feng J., Abbasi S., Atsumi T., Morse H.C. 3rd IRF8 regulates B-cell lineage specification, commitment, and differentiation. Blood. 2008; 112:4028–4038. 18799728 10.1182/blood-2008-01-129049 PMC2581985

[30] Vinogradov A.E. , Anatskaya O.V. DNA helix: the importance of being AT-rich. Mamm. Genome. 2017; 28:455–464. 28836096 10.1007/s00335-017-9713-8

[31] Zhou Y. , Liang Y., Lynch K.H., Dennis J.J., Wishart D.S. PHAST: a fast phage search tool. Nucleic Acids Res. 2011; 39:W347–352. 21672955 10.1093/nar/gkr485 PMC3125810

