# Supplementary material for: Multiplex indexing approach for the detection of DNase I hypersensitive sites in single cells
Source: Nucleic Acids Res. 2021 Mar 8;49(10):e56. doi: 10.1093/nar/gkab102 (PMC8191781; doi:10.1093/nar/gkab102)
Supplement: gkab102_Supplemental_Files [file gkab102_supplemental_files.zip › Supplementary_figures.pdf]

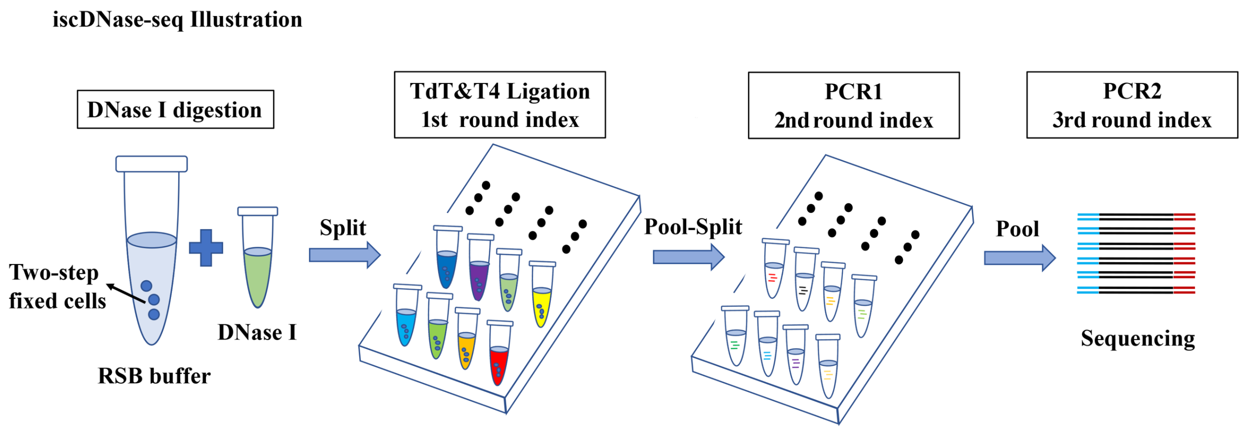

Supplementary Figure 1. Illustration of iscDNase-seq methods. Experimental flow chart of the iscDNase-seq protocol.

TdT&T4 ligation Illustration

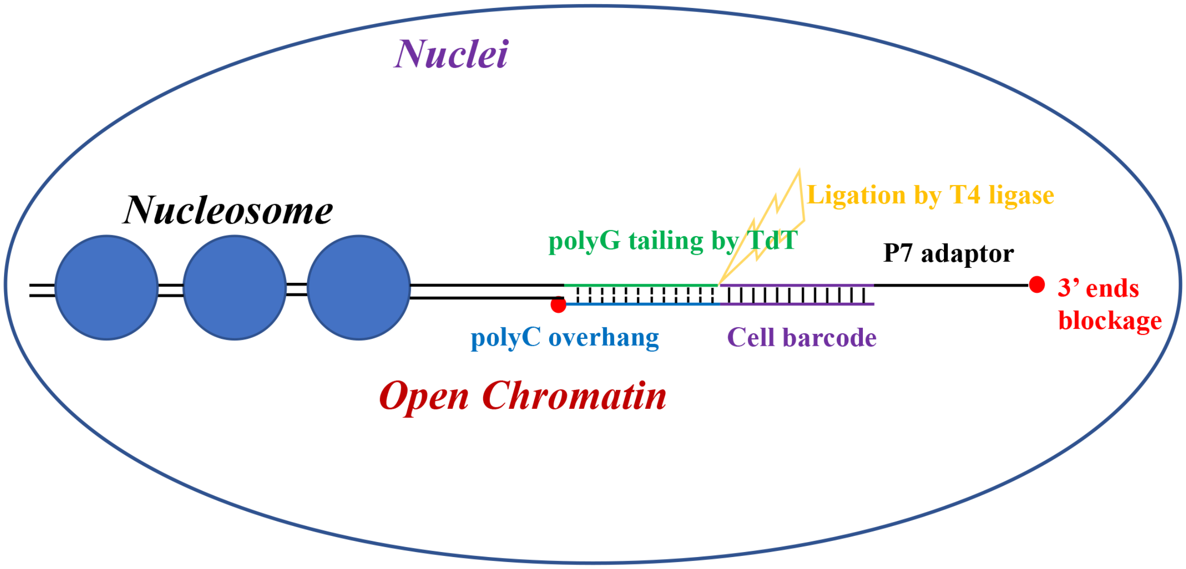

Supplementary Figure 2. Illustration of TdT&T4 Ligation strategy. The sequence of reaction is as following: (1) addition of several dGs to the 3' end of DNA by TdT; (2) annealing of oligo-dC barcode primer to the oligo dG sequence; (3) repairing the oligo-dG and T7 adaptor sequences by T4 DNA ligase.

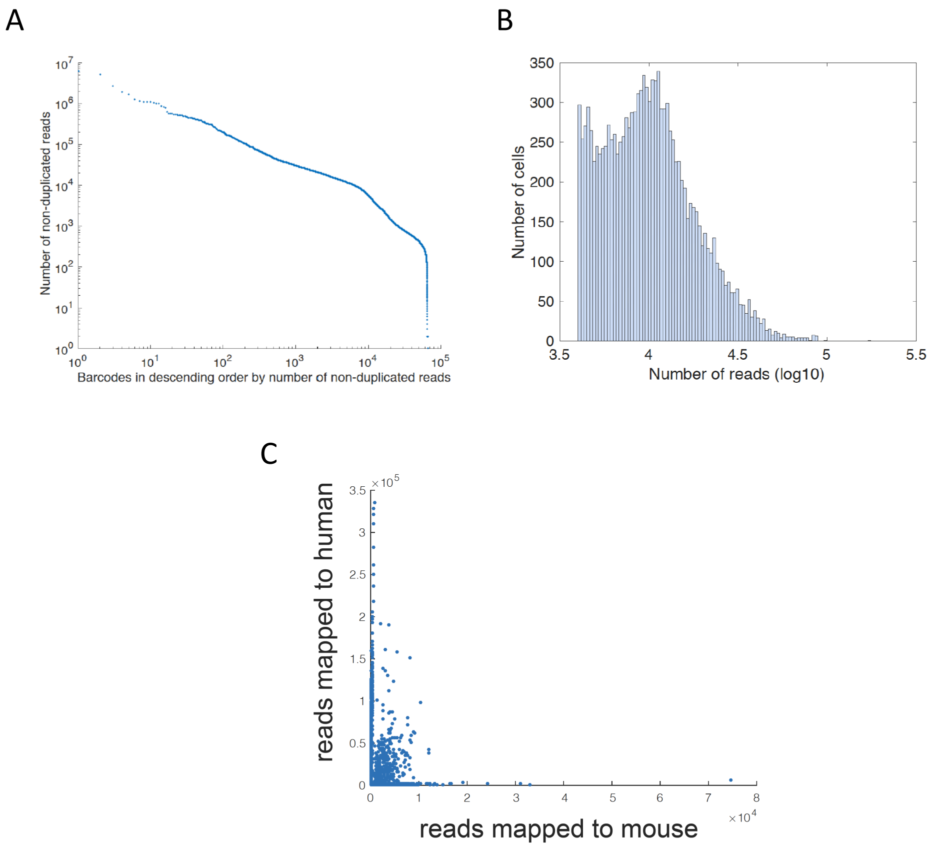

Supplementary Figure 3. Quality control of the iscDNase-seq.

A. A knee plot for the iscDNase-seq single cell data.

B. A distribution plot for the reads per cell in which reads is in the log10 scale.

C. Human and mouse cells were mixed before the DNase I digestion step. Following the library construction and sequencing, the numbers of sequence reads mapped to either the human (y-axis) and mouse (x-axis) genomes from each single cell were plotted. Each dot represents one barcode.

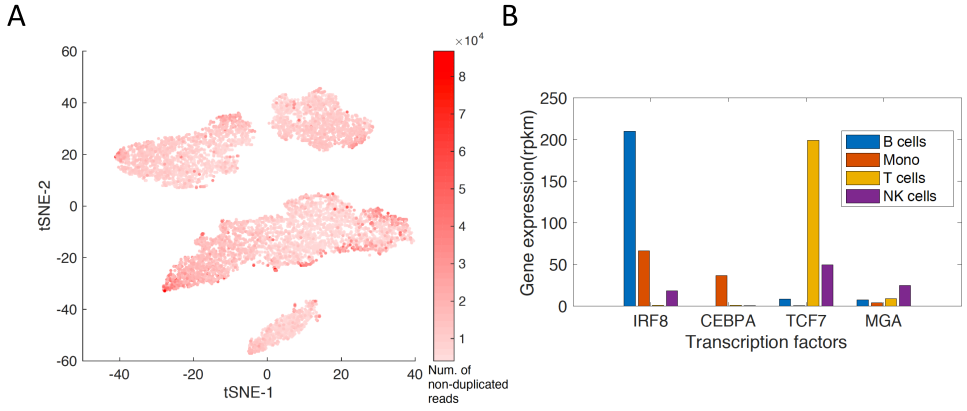

Supplementary Figure 4. Sequencing depth in each cell and TF Motifs enriched in clusters.

A. A t-SNE visualization of cells with the number of non-duplicated reads.

B. Bar plot showing the gene expression (rpkm) in monocytes, T cells, B cells, and NK cells for selected TFs. IRF8, CEBPA, TCF7, MAG were selected.

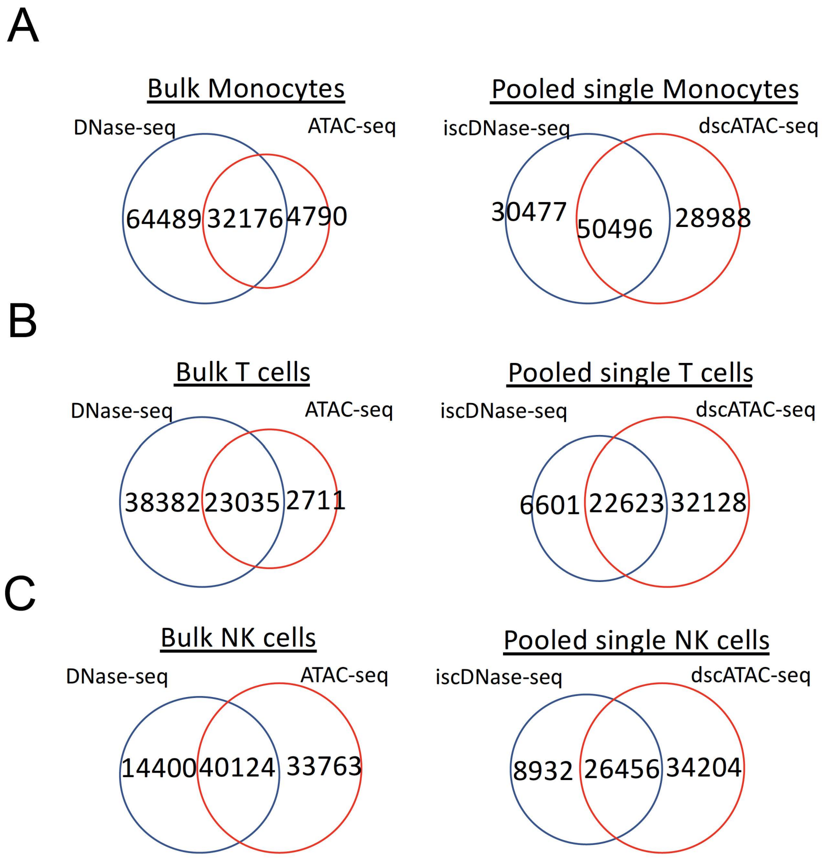

Supplementary Figure 5. Venn diagrams between iscDNase-seq and dscATAC-seq for T cells, NK cells and monocytes (right). Venn diagrams between bulk cell DNase-seq and ATAC-seq for T cells, NK cells and monocytes (left).

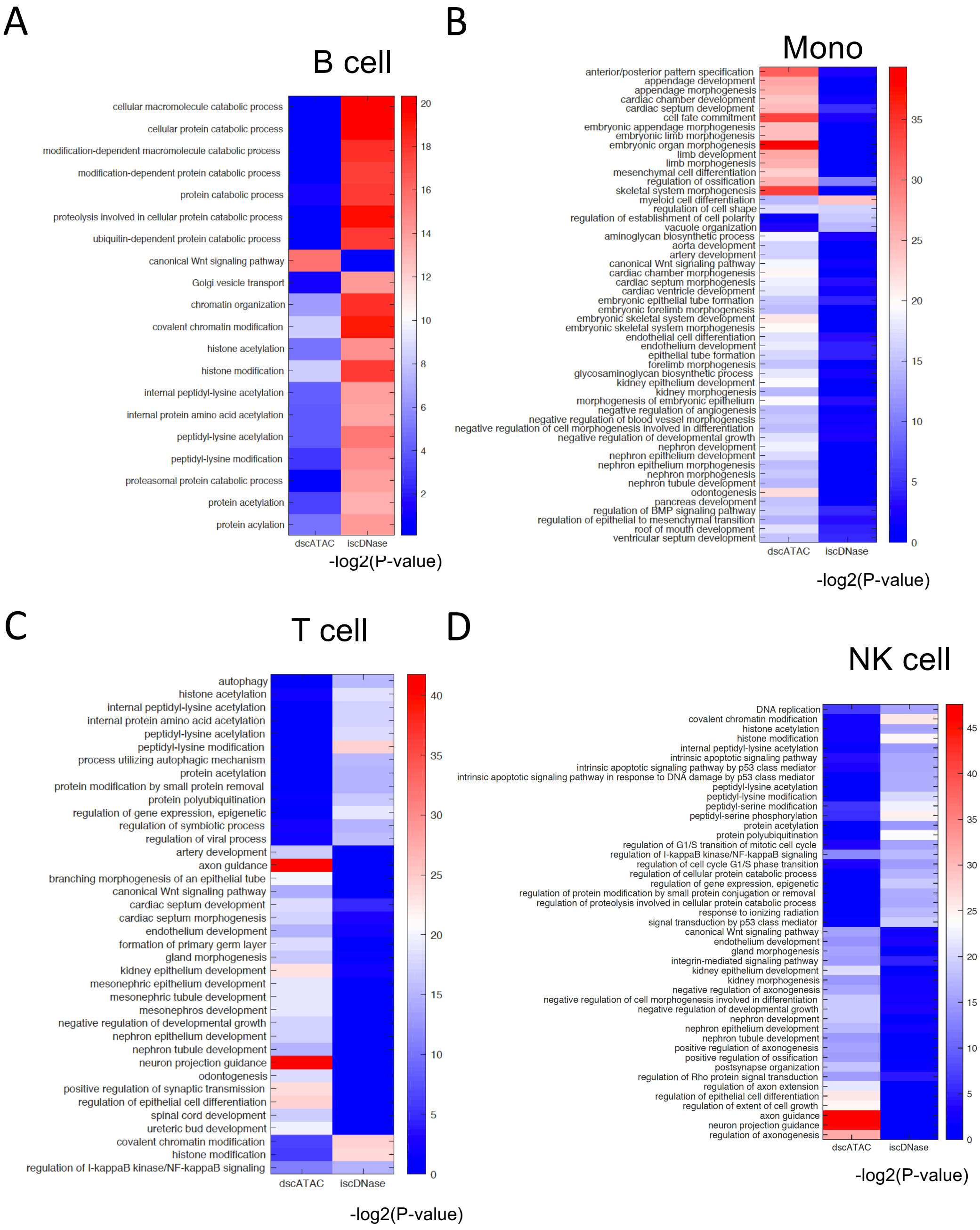

Supplementary Figure 6. Gene ontology analysis for the unique iscDNase-seq peaks and unique dscATAC-seq peaks. The four heatmaps are for (A) B cells, (B) monocytes, (C) T cells, and (D) NK cells.

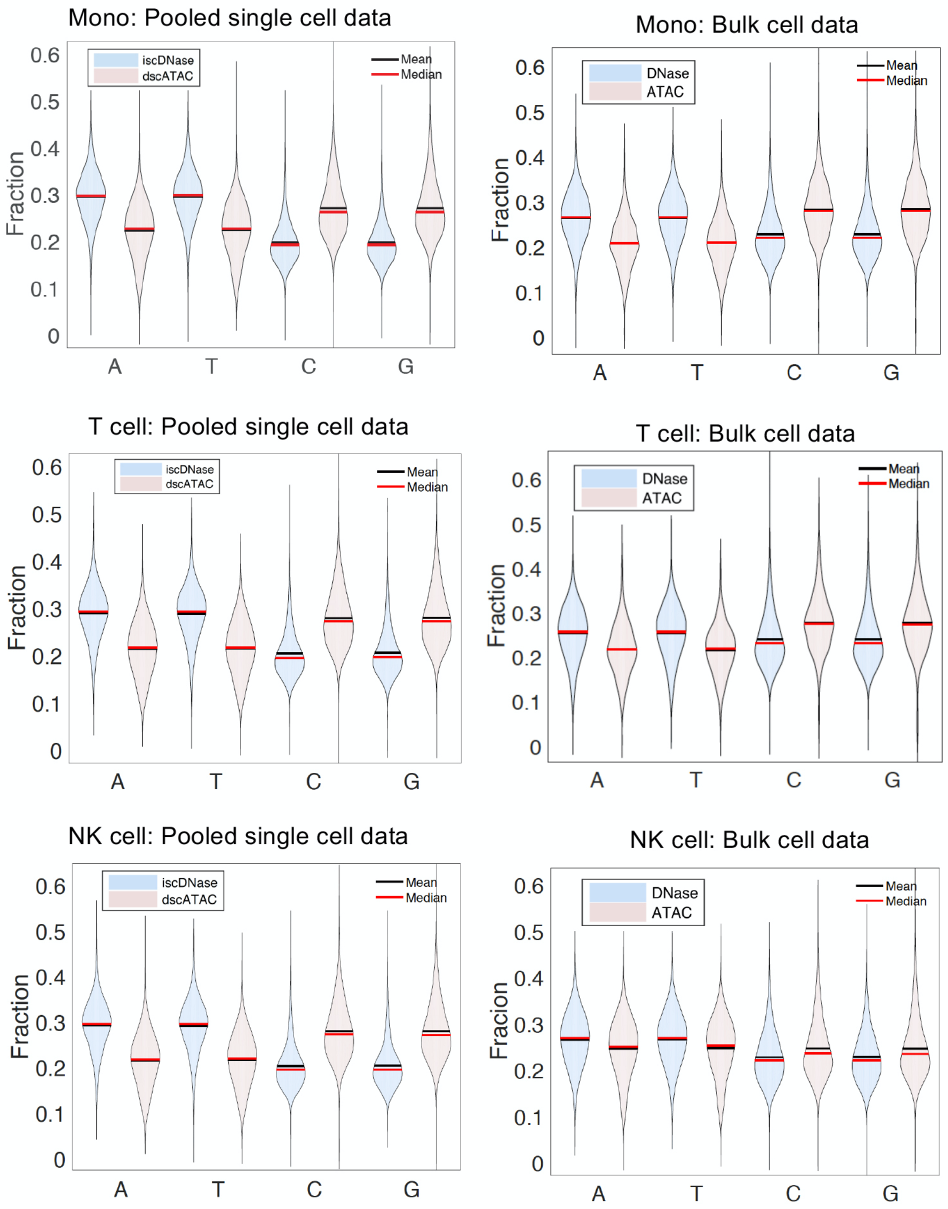

Supplementary Figure 7. Violin plots showing the fraction of nucleotides (A, T, C, and G) for iscDNase-seq and dscATAC-seq (left). Violin plots showing the fraction of nucleotides (A, T, C, and G) for bulk cell DNase-seq and bulk cell ATAC-seq (right).

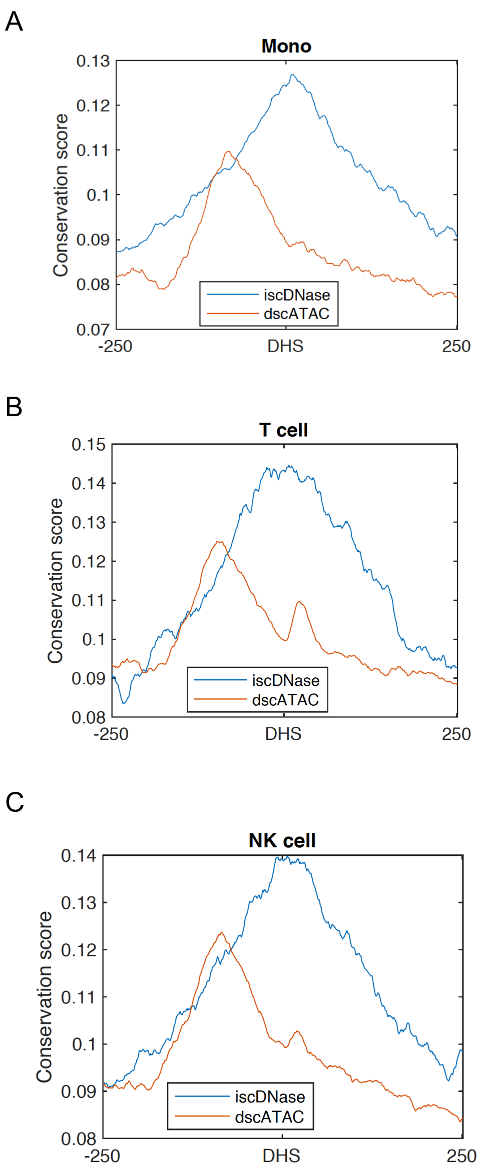

Supplementary Figure 8. Sequence conservation score plot for unique iscDNase-seq and unique dscATAC-seq peaks for (A) Monocytes, (B) T cells, and (C) NK cells.

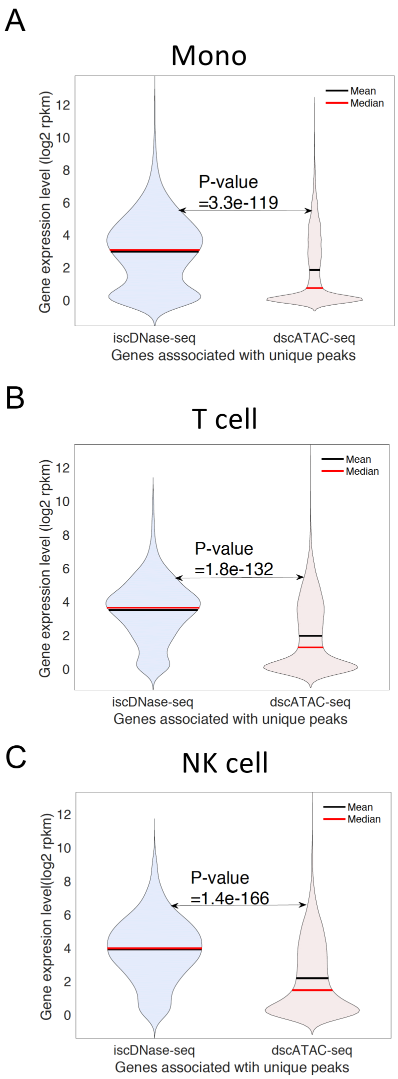

Supplementary Figure 9. Violin plots showing the gene expression levels for genes associated with the unique iscDNase-seq peaks and unique dscATAC-seq peaks for (A) Monocytes, (B) T cells, and (C) NK cells.

Supplementary Table 1. Reference data from bulk DNase-seq, ATAC-seq, and RNA-seq.

Supplementary Table 1. Reference data index.

Name	Use	ID	Source
Human CD19 cells (dscATAC-seq)	dscATAC-seq	GSE123578	GEO
Human NK cells (dscATAC-seq)	dscATAC-seq	GSE123578	GEO
Human CD4 T cells (dscATAC-seq)	dscATAC-seq	GSE123578	GEO
Human monocytes (dscATAC-seq)	dscATAC-seq	GSE123578	GEO
B cell	Bulk DNase-seq	ENCFF566GSP	ENCODE
CD4-positive alpha-beta T cell	Bulk DNase-seq	ENCFF041CSN	ENCODE
CD14-positive monocyte	Bulk DNase-seq	ENCFF001DZH	ENCODE
NK cells	Bulk DNase-seq	ENCFF384FJC	ENCODE
NK cells	Bulk DNase-seq	ENCFF505OFY	ENCODE
Bulk B	Bulk ATAC-seq	SRR7650771	GEO
Effector CD4pos T	Bulk ATAC-seq	SRR7650788	GEO
Monocyte	Bulk ATAC-seq	SRR7650886	GEO
Immature NK	Bulk ATAC-seq	SRR7650763	GEO
mature NK	Bulk ATAC-seq	SRR7650764	GEO
Memory NK	Bulk ATAC-seq	SRR7650766	GEO
Human T cells	RNA-seq	GSM1256828	GEO
Human T cells	RNA-seq	GSM1256829	GEO
Human B cells	RNA-seq	GSM1256812	GEO
Human B cells	RNA-seq	GSM1256813	GEO
Human B cells	RNA-seq	GSM1256814	GEO
Human B cells	RNA-seq	GSM1256815	GEO
Human B cells	RNA-seq	GSM1256816	GEO
Human monocytes	RNA-seq	GSM1256822	GEO
Human monocytes	RNA-seq	GSM1256823	GEO
Human monocytes	RNA-seq	GSM1256824	GEO
Human monocytes	RNA-seq	GSM1256825	GEO
Human monocytes	RNA-seq	GSM1256826	GEO
Human NK cells	RNA-seq	GSM1982286	GEO
Human NK cells	RNA-seq	GSM1982287	GEO
Human NK cells	RNA-seq	GSM1982293	GEO
Human CD19 B cells	scRNA-seq		10x
Human CD14 Monocytes	scRNA-seq		10x
Human CD4 Helper T cells	scRNA-seq		10x
Human CD56 NK cells	scRNA-seq		10x
phastCons17way	Conservation annotations	-	UCSC

Supplementary Material S1. Information of Barcodes.

Supplementary Material S2. A list of reagents.

Supplementary Material S3. Single cell reads statistics
