# Supplementary material for: Multiplex indexing approach for the detection of DNase I hypersensitive sites in single cells
Source: Nucleic Acids Res. 2021 Mar 8;49(10):e56. doi: 10.1093/nar/gkab102 (PMC8191781; doi:10.1093/nar/gkab102)
Supplement: gkab102_Supplemental_Files [file gkab102_supplemental_files.zip › Supplementary_material2.docx]

**Reagents**

**Critical:** All reagents must be kept DNase-free.

- Nuclease-free water (Life Technologies, cat. #AM9930)
- 1M Tris-HCl (pH 7.5; K-D Medical, cat. #RGF-3340)
- 1M Tris-HCl (pH 8.0; K-D Medical, cat. #RGF-3360)
- Triton X-100 (Quality Biological, cat. #A611-M143-13)
- 2M Calcium chloride (Quality Biological, cat. #351-130-721EA)
- EDTA
- EGTA, Molecular Biology Grade (Quality Biological, cat. #A611-0732-88)
- 5M Sodium Chloride, Molecular Biology Grade (Promega, cat. #V4221)
- 3M Sodium Acetate pH5.2, Molecular Biology Grade (Quality Biological, cat. #351-035-721)
- culture medium DMEM (Invitrogen cat. #10566-016)
- FBS (Sigma cat. #F4135-500ML)
- DSG (ThermoFisher Scientific, cat. #20593)
- Pierce™ 16% Formaldehyde (w/v), Methanol-free (ThermoFisher Scientific, cat. #28906)
- Glycine (Sigma cat. #50046-250G)
- 10% SDS (Quality Biological, cat. #351-032-101)
- Proteinase K, recombinant, PCR grade (Sigma Aldrich, cat. #3115836001)
- Phenol–chloroform, pH 6.7/8.0 (Amresco, cat. no. 0883)
- Glycogen, molecular-biology-grade (Sigma-Aldrich, cat. no. 10901393001)
- DNase I recombinant (DNase) (Sigma-Aldrich, cat. no. 4536282001)
- Micrococcal Nuclease (MNase) (Sigma, Cat. #N3755-500UN)
- T4 Polynucleotide Kinase (New England BioLabs, cat. #M0201L)
- Terminal Transferase (New England BioLabs, cat. #M0315L)
- T4 DNA ligase (400,000U/ml) (New England BioLabs, cat. #M0202S)
- Adenosine 5'-Triphosphate (ATP) (New England BioLabs, cat. #P0756S)
- Deoxynucleotide (dNTP) Solution Set (New England BioLabs, cat. #N0446S)
- Phusion^®^ High-Fidelity PCR Master Mix with HF Buffer (New England BioLabs, cat. #M0531S)
- MinElute^®^ Reaction Cleanup Kit (Qiagen, cat. #28206)
- EB buffer (Qiagen, cat. #19086)
- Klenow Fragment (3'→5' exo-) (5000 units/ml) (New England BioLabs, cat. #M0212S)
- Exonuclease I (ThermoFisher Scientific, cat # EN0582)
- End-It™ DNA End-Repair Kit (Epicentre, cat #ER0720)
- dATP (10mM) (ThermoFisher Scientific, cat #18252015)
- NEBuffer 2 (New England BioLabs, cat #B7002S)
- MinElute Gel Extraction Kit (Qiagen, cat. #28604)

**Equipment**

- 0.2ml PCR tubes (Molecular Bio-Products, cat. #3418)
- LightCycler^®^ 480 Multiwell Plate 96, white (Roche, cat. #04729692001)
- LightCycler^®^ 480 Sealing Foil (Roche, cat. #04729757001)
- Micro tube 1.5ml DNA LowBind (Sarstedt, cat. #72.706.700)
- Falcon^®^ Round Bottom Polystyrene Test Tube with Blue Cell Strainer Snap Cap (Corning, cat. #352235)
- Tabletop microcentrifuge (VWR MicroStar 17R, cat. #521-1647)
- Sorvall Legend RT Refrigerated Benchtop Centrifuge (Marshall scientific)
- MJ Research PTC-200 Thermal Cycler (MJ Research, cat. #8252-30-0001)
- E-Gel^®^ EX Agarose Gels Starter Kit, 2% (Invitrogen, cat #G6512ST)
- E-Gel^®^ EX Agarose Gels, 2% (Invitrogen, cat #G401002)
- E-Gel^®^ 1 Kb Plus DNA Ladder (Invitrogen, cat #10488090)
- Freezers, −20 °C and −80 °C (Liebherr cat. #GPESF1476)
- Qubit fluorometer (Life Technologies, cat. #Q32866)
- BD FACSAria II (BD Biosciences)
- HiSeq 3000 (Illumina)
